# Sclerosing Mesenteritis: A Diagnostic Challenge Between Benignity and Malignancy

**DOI:** 10.7759/cureus.95567

**Published:** 2025-10-28

**Authors:** Joana Filipa Carneiro de Moura, Ana Rua

**Affiliations:** 1 General Medicine, Unidade Local de Saúde do Tâmega e Sousa, Porto, PRT; 2 Family Medicine, Unidade Local de Saúde do Tâmega e Sousa, Porto, PRT

**Keywords:** chronic abdominal pain, differential diagnosis, mesenteric mass, mesenteric panniculitis, multidisciplinary approach

## Abstract

Sclerosing mesenteritis is a rare, benign, fibroinflammatory disorder of the mesentery that often presents radiographic features that could be mistaken for malignancy. This case study report describes the diagnostic and therapeutic journey of a woman in her early forties who had ongoing abdominal pain, diarrhea, and weight loss. A retractile fibrosing mesenteric mass with vascular compromise was discovered by imaging. Sclerotic mesenteritis was confirmed by histology. Subsequent analyses excluded malignant, autoimmune, and infectious causes. For managing symptoms, a three-month course of oral budesonide, agents such as polyethylene glycol, and a dietary plan were provided. Suspicions of superior mesenteric artery syndrome were later raised by vascular involvement. Sclerosing mesenteritis is a challenging diagnosis, associated with several benign and malignant etiologies. In this case, the symptoms may result from mass effect or mask another pathology, but the extensive study conducted was insufficient to allow a definitive diagnosis.

## Introduction

Sclerosing mesenteritis is an uncommon, non-neoplastic inflammatory condition characterized by chronic inflammation, fat necrosis, and fibrosis of the mesenteric adipose tissue. Although its prevalence is estimated at approximately 0.6% in abdominal computed tomography (CT) scans, the disease remains underdiagnosed due to its nonspecific clinical presentation and often incidental radiological findings [1,2]. It primarily affects middle-aged to older Caucasian men, although it can occur in individuals of any age or sex [3].

The etiology of sclerosing mesenteritis is incompletely understood. Proposed contributing factors include prior abdominal surgery or trauma, autoimmune mechanisms, infections, ischemia, and paraneoplastic processes [2-4]. In some cases, no identifiable cause is found, and the condition is considered idiopathic. Histologically, sclerosing mesenteritis represents a spectrum of three overlapping entities: mesenteric lipodystrophy (fat necrosis), mesenteric panniculitis (inflammatory cell infiltration), and retractile mesenteritis (fibrosis and mass effect) [1,5].

Clinical manifestations are highly variable, ranging from asymptomatic incidental findings to abdominal pain, nausea, vomiting, altered bowel habits, and systemic features such as weight loss or low-grade fever [1,6]. In rare cases, the mass effect from mesenteric fibrosis may lead to complications such as bowel obstruction or vascular compression [4,7].

Radiological imaging is highly important for initial detection, often revealing a mesenteric soft-tissue mass with fat stranding around mesenteric vessels [5,6]. However, these features are not pathognomonic, and sclerosing mesenteritis can mimic malignancies such as lymphoma or carcinoid tumors. Definitive diagnosis frequently requires histopathological confirmation, although tissue sampling may be limited by the lesion's location or fibrosis [2,6].

While many cases are asymptomatic or self-limiting, sclerosing mesenteritis can have a prolonged and debilitating course. Studies indicate that approximately 17% of patients experience fatal outcomes related to the disease or its treatment. A systematic review of 192 cases reported 14 deaths, with 12 (85.7%) attributed to complications of sclerosing mesenteritis. The median survival duration for patients with symptomatic disease is around 10 months, with an all-cause mortality rate of up to 7.3% [8].

Awareness of sclerosing mesenteritis is crucial for clinicians, as it should be considered in the differential diagnosis of unexplained abdominal pain or mesenteric masses. Radiological findings, although suggestive, are often nonspecific, underscoring the importance of histopathological confirmation. Management must be individualized according to symptom severity and disease progression, ranging from observation in asymptomatic cases to immunosuppressive therapy in patients with significant symptoms or progressive disease. Clinicians should also remain vigilant for potential complications such as bowel obstruction or vascular compromise, which require timely intervention.

## Case presentation

A woman in her early forties, with no significant personal or family medical history and no regular medication use, began experiencing symptoms in September 2019. Over the course of one year, she reported multiple episodes of colicky abdominal pain, rated 6 out of 10 in intensity, with no clear relief from changes in position. Additional symptoms included early satiety, postprandial heartburn, and an unintentional weight loss of approximately 5 kg. She also noted occasional night sweats. Bowel habits fluctuated between episodes of diarrhea and constipation, although she denied nausea or vomiting.

Physical examination revealed a soft, non-distended abdomen with no palpable masses. Mild hepatomegaly was observed. Deep palpation of the right upper quadrant elicited pain, and there was mild tenderness in the remaining quadrants.

The patient presented to the local emergency department on multiple occasions, typically with complaints of abdominal discomfort accompanied by variable bowel habits. Initial diagnoses included presumed acute gastroenteritis, and she was managed symptomatically with probiotics. Due to persistent right upper quadrant pain lasting over one year, she was referred to her primary care physician in September 2020 for further investigation.

Investigations

**Figure 1:**
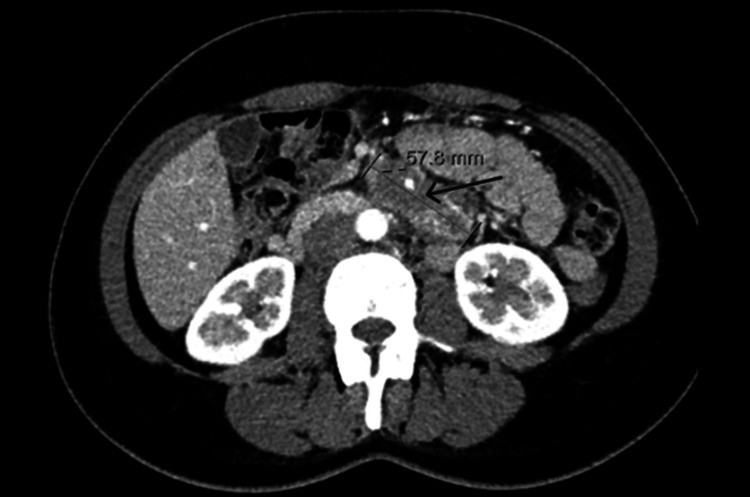
Contrast-enhanced abdominal CT scan, taken in January 2021, showcasing a hypodense mass centered in the mesentery (indicated by the arrow), encasing mesenteric arterial branches at this level, with relatively coarse calcifications within. The lesion measures approximately 60 × 21 mm in its maximal axial dimensions, with a longitudinal extension of about 50 mm. Imaging features are suggestive, as a primary diagnostic consideration, of sclerosing mesenteritis. CT: computed tomography

Initial clinical evaluation raised the possibility of a functional bowel disorder. However, due to the persistence of symptoms, an abdominal ultrasound was performed in November 2020, which revealed retroperitoneal lymphadenopathy. A subsequent abdominal CT scan, requested in primary care and illustrated in Figure 1, identified a soft-tissue mass at the mesenteric root, anterolaterally to the major abdominal vessels. The lesion appeared to represent a conglomerate of lymph nodes, with scattered fine calcifications clustered in the inferior and left aspects of the mass. The lesion extended from the region immediately below the cephalopancreatic junction to the level of the lower poles of the kidneys, occupying the mid-abdominal compartment. It measured approximately 60 mm in the craniocaudal axis and 56 × 27 mm in the axial plane. Laboratory studies revealed no abnormalities, including normal hepatic and renal function parameters, and tumor marker levels were all within reference ranges (carcinoembryonic antigen and CA 19-9). The patient was subsequently referred to tertiary care for further evaluation of a suspected mesenteric mass.

To facilitate histopathological characterization of the mesenteric lesion, an endoscopic ultrasound was performed in January 2021. This revealed a heterogeneous, predominantly hypoechoic mass with poorly defined margins, adjacent to the second portion of the duodenum. The lesion exhibited significant internal vascularization and maintained a close anatomical relationship with the superior mesenteric artery (SMA), although no radiological evidence of invasion into adjacent structures was observed.

A fine-needle aspiration biopsy was subsequently performed, yielding a representative sample of fibrous tissue. Due to the limited quantity and nature of the material, further histological delineation was not feasible. A second endoscopic ultrasound-guided biopsy was performed in February 2021, yielding a more substantial tissue sample. Histopathological evaluation at that time suggested features consistent with a lymphoproliferative disorder. Given the diagnostic uncertainty, the patient underwent a multidisciplinary assessment involving specialists in internal medicine, hematology, gastroenterology, and general surgery. In March 2021, a laparoscopic biopsy was performed, which confirmed fibroinflammatory changes consistent with sclerosing mesenteritis.

Due to the persistence of symptoms, including diffuse abdominal pain, episodes of diarrhea occurring up to six to seven times per day alternating with constipation, and the intermittent appearance of cutaneous lesions, further gastrointestinal evaluation was pursued. Upper gastrointestinal endoscopy and colonoscopy were performed and demonstrated no pathologic abnormalities, except for chronic gastritis associated with Helicobacter pylori infection. Treatment with oral budesonide at a dosage of 9 mg daily over three months resulted in partial improvement of gastrointestinal symptoms.

In the latter part of 2021, PET-CT demonstrated diffuse fluorodeoxyglucose uptake within the nasopharynx and palatine tonsils, in addition to bilateral upper laterocervical lymphadenopathy exhibiting mild metabolic activity. These findings were interpreted as consistent with an inflammatory process. Subsequent referral to an otorhinolaryngologist was made, and histological analysis of nasopharyngeal and tonsillar biopsies revealed reactive lymphoid hyperplasia, thereby excluding neoplastic involvement. No evidence of lymphoproliferative disease was identified upon extended otolaryngological evaluation.

A comprehensive immunological and hematological workup was also conducted, including erythrocyte sedimentation rate (ESR), C-reactive protein (CRP), antinuclear antibody (ANA) testing, extractable nuclear antigen (ENA) panel, rheumatoid factor, anti-neutrophil cytoplasmic antibodies (ANCA), serum immunoglobulin quantification (including subclass IgG4), and peripheral blood immunophenotyping. All parameters were within normal reference ranges, as summarized in Table 1.

**Table 1:** Laboratory findings in 2021 ESR: erythrocyte sedimentation rate, CRP: C-reactive protein, ALT: alanine aminotransferase, ALT: alanine aminotransferase, ALP: alkaline phosphatase, LDH: lactate dehydrogenase, ACE: angiotensin-converting enzyme, CEA: carcinoembryonic antigen, CA 19-9: carbohydrate antigen 19-9, A/G ratio: albumin-to-globulin ratio, IgG4: immunoglobulin G subclass 4, IgG: immunoglobulin G, IgA: immunoglobulin A, IgM: immunoglobulin M, tTG: tissue transglutaminase, EMA: endomysial antibodies, DGP: deamidated gliadin peptide

Test	Result	Reference range	Interpretation
ESR	25 mm/h	<20 mm/h	Mildly elevated; suggests inflammation
CRP	6.3 mg/L	<5.0 mg/L	Elevated; supports the inflammatory process
Serum IgG4	56 mg/dL	<135 mg/dL	Normal; argues against IgG4-related disease
Serum IgG (total)	1320 mg/dL	700-1600 mg/dL	Normal
Complete blood count	Within normal limits	-	No abnormalities detected
Hemoglobin	13.4 g/dL	12-16 g/dL	Normal
White blood cell count	6.8 ×10⁹/L	4.0-10.0 ×10⁹/L	Normal
Platelet count	290 ×10⁹/L	150-400 ×10⁹/L	Normal
ALT	25 U/L	7-56 U/L	No hepatic dysfunction
ALT	25 U/L	7-56 U/L	No hepatic dysfunction
ALP	78 U/L	45-120 U/L	No hepatic dysfunction
Creatinine	0.8 mg/dL	<1.2 mg/dl	Normal renal function
Serum albumin	4.1 g/dL	3.5-5.0 g/dL	Normal
Serum calcium	9.4 mg/dL	8.5-10.5 mg/dL	Normal
LDH	180 U/L	135-225 U/L	Normal
Autoimmune panel (ANA, ANCA, RF, anti-CCP)	Negative	-	No evidence of systemic autoimmune disease
Serum ACE	35 U/L	8-52 U/L	Normal; argues against sarcoidosis
Tumor markers (CEA, CA 19-9)	Within normal limits	-	No evidence of neoplastic process
Beta-2 microglobulin	2000 µg/L	1090-2530 µg/L	Normal
Total serum protein	82.9 g/L	64-83 g/L	Upper-normal; mild hyperproteinemia
Albumin	50.30%	49.7-64.4%	Normal
Alpha-1 globulin	4.20%	4.8-10.1%	Slightly decreased
Alpha-2 globulin	9.20%	8.5-15.1%	Normal
Beta globulin	13.00%	7.8-13.1%	Normal
Gamma globulin	15.70%	10.5-19.5%	Normal
A/G ratio	1.01	0.99-1.81	Normal
Serum immunoglobulins		-	
IgG	1450 mg/dL	650-1500 mg/dL	Normal
IgA	304 mg/dL	78-312 mg/dL	Normal
IgM	187 mg/dL	55-300 mg/dL	Normal
Kappa free light chains	432mg/dL	200-440 mg/dL	Normal
Lambda free light chains	223 mg/dL	110-240 mg/dL	Normal
Kappa/lambda ratio	2.09	1.35-2.65	Normal; argues against monoclonal gammopathy
Flow cytometry (peripheral blood)			Normal immunophenotype; no evidence of lymphoproliferative disorder
CD2+	95.00%	-	T-cell population within expected limits
CD3+	81.70%	-	Predominant T-cell subset
CD3+TCRα/β+	89.30%	-	Normal T-cell receptor expression
CD3+TCRγ/δ+	10.70%	-	Within normal range
CD3+CD4+	60.70%	-	Normal CD4 population
CD4+/CD8+	1.30%	-	Normal ratio
CD3+CD8+	31.90%	-	Normal cytotoxic subset
CD16/56+ (NK-cells)	13.30%	-	Normal NK-cell proportion
CD19+ (B-cells)	5.00%	-	Normal B-cell percentage
B-cell light chains	Kappa 59%, Lambda 41%	-	Polyclonal B-cell population (no clonal restriction)
Anti-tTG IgA	<2 U/mL	<10 U/mL	No serologic evidence of celiac disease
Anti-EMA	Negative	Negative	No serologic evidence of celiac disease
Anti-DGP IgG	<10 U/mL	<10 U/mL	No serologic evidence of celiac disease

In April 2021, the patient reported a slight improvement in gastrointestinal symptoms. However, in September 2021, she developed cutaneous eruptions, which resolved spontaneously. These lesions were clinically nonspecific and were presumed to be of allergic origin. Although the presentation was not suggestive of carcinoid syndrome, this differential diagnosis was formally excluded. In this context, a dermatological consultation was arranged. The evaluating dermatologist concurred with a likely allergic etiology. During the examination, a pigmented cutaneous lesion of concern was identified and subsequently biopsied. Histopathological analysis confirmed a benign lentiginous junctional nevus.

In 2022, serial PET-CT imaging continued to demonstrate metabolic findings consistent with persistent inflammatory activity. An abdominal CT enterography revealed a slight reduction in the mesenteric mass (now approximately 51 mm), in keeping with a fibrosing presentation of mesenteric panniculitis. The imaging also revealed established collateral venous circulation secondary to occlusion of the superior mesenteric vein (SMV).

**Figure 2:**
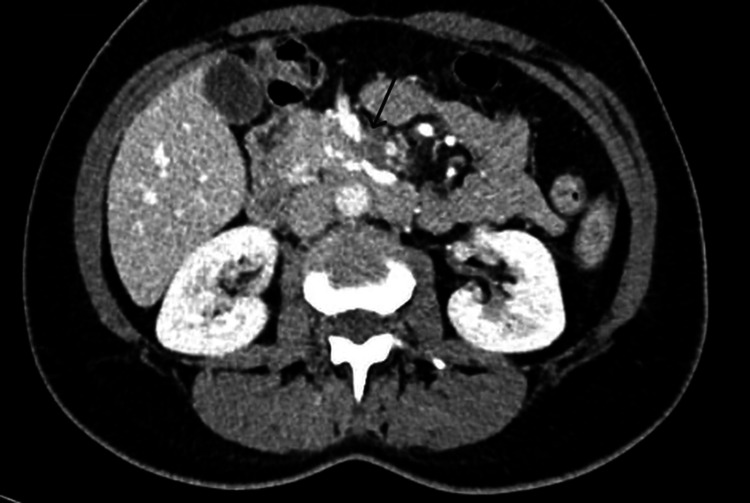
Contrast-enhanced CT scan from November 2024 showcasing an area of infiltrative densification (indicated by the arrow) with a mass-like appearance along the root of the mesentery, involving the adjacent vascular structures, measuring approximately 5 × 2.5 cm. It caused occlusion of a segment of the SMV, with visualization of some ectatic venous structures draining into the proximal segment of the vein, predominantly in the right abdominal quadrants. CT: computed tomography, SMV: superior mesenteric vein

In November 2024, following an episode of functional gastrointestinal symptoms, a repeat abdominal CT confirmed the persistence of the fibrosing mesenteric mass and reaffirmed occlusion of the SMV, as shown in Figure 2. Imaging revealed an area of infiltrative mesenteric densification with a mass-like configuration extending from the mesenteric root and involving adjacent vascular structures. The lesion measured approximately 5.0 × 2.5 cm in axial dimensions and contained scattered internal calcifications. Histological confirmation of sclerosing mesenteritis had previously been established. The lesion was noted to cause segmental occlusion of the SMV, with collateral venous drainage via ectatic vessels observed predominantly within the right abdominal quadrants.

In 2025, due to worsening postprandial abdominal pain and imaging findings suggestive of vascular compromise, a clinical suspicion of SMA syndrome (Wilkie’s syndrome) was raised. A multidisciplinary assessment was initiated, involving specialists in gastroenterology, general surgery, and vascular surgery.

In June 2025, the vascular surgery team concluded that fibrotic retraction was impairing perfusion through the SMA. Although a definitive surgical strategy has not yet been finalized, proposed interventions include endovascular stenting of the SMA or surgical bypass to restore mesenteric blood flow and relieve the patient’s symptoms. The patient remains under active vascular surveillance and is receiving symptomatic management pending further diagnostic clarification and interdepartmental deliberation.

Treatment

Initial therapeutic management consisted of oral budesonide 9 mg daily for three months, resulting in partial symptomatic improvement. To address associated constipation and colicky abdominal pain, the patient was prescribed macrogol (13.125 g once daily) and oral butylscopolamine (10 mg, up to three times per day). Concurrently, dietary modifications were implemented to support gastrointestinal function.

A concurrent Helicobacter pylori infection was identified and treated successfully with standard eradication therapy. Cyclical courses of corticosteroids, each lasting three months, were administered, resulting in partial relief of abdominal discomfort. In November 2023, a 15-day course of oral rifaximin (500 mg twice daily) was introduced to manage diarrheal episodes, with modest clinical improvement.

Patient-reported outcomes indicated improvement in quality of life, particularly regarding abdominal pain and bowel habits. Pain scores, assessed on a 0-10 numerical rating scale, decreased from 6 to 2-3 following this regimen and remained stable thereafter, reflecting moderate improvement in symptom severity. Throughout the treatment period, inflammatory markers, notably CRP, consistently remained mildly elevated, oscillating between 6 and 8 mg/L (normal <5 mg/L), reflecting persistent low-grade inflammation.

In November 2024, the exacerbation of gastrointestinal symptoms, in conjunction with progressive radiological findings, necessitated referral to vascular surgery in 2025 for assessment of suspected SMA compression. The vascular surgery team evaluated the patient, and a contrast-enhanced CT angiography is planned to assess vascular anatomy and determine whether an endovascular stent or an open surgical bypass is the most appropriate intervention.

Outcome and follow-up

**Figure 3:**
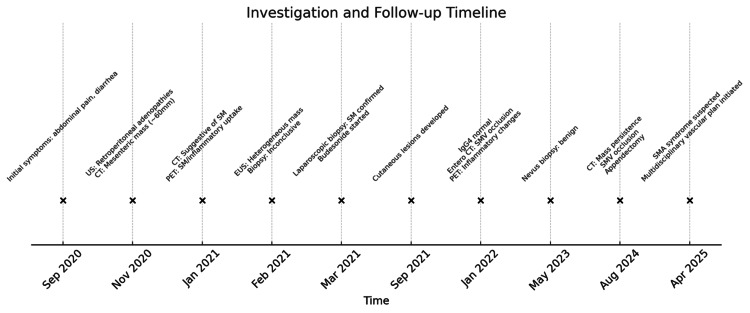
Investigation and follow-up timeline US: ultrasound, CT: computed tomography, PET: positron emission tomography, SM: sclerosing mesenteritis, EUS: endoscopic ultrasound, IgG4: immunoglobulin G subclass 4, SMV: superior mesenteric vein, SMA: superior mesenteric artery

Over five years, the patient exhibited persistent gastrointestinal symptomatology accompanied by progressive radiological evidence of vascular involvement. Imaging studies conducted in 2025 demonstrated stability in the dimensions of the mesenteric mass; however, they revealed increased SMA compression. The patient remains under ongoing surveillance by the vascular surgery team, with potential interventional management currently under consideration. The follow-up and diagnostic workup are summarized in the timeline presented in Figure 3.

## Discussion

The initial differential diagnosis encompassed malignant, autoimmune, infectious, and fibrosing entities, including lymphoma, carcinoid tumor, IgG4-related disease, tuberculosis, sarcoidosis, and retroperitoneal fibrosis. Lymphoma was considered a primary differential diagnosis; however, it was subsequently excluded based on the absence of systemic lymphadenopathy, non-suggestive PET-CT findings, and negative histopathological analysis. A carcinoid tumor was deemed unlikely due to the lack of clinical features characteristic of carcinoid syndrome, normal neuroendocrine biomarkers, and non-diagnostic imaging studies.

IgG4-related disease was ruled out due to normal serum IgG4 concentrations and the absence of typical histological features. Granulomatous diseases, including tuberculosis and sarcoidosis, were excluded based on negative microbiological screening and the absence of granulomatous inflammation on tissue specimens. Retroperitoneal fibrosis remained a consideration; however, the central mesenteric location of the lesion, in conjunction with the absence of characteristic findings such as ureteric or aortic involvement, made this diagnosis less likely. Furthermore, the observed histopathological pattern and the lack of systemic manifestations typically associated with these conditions further diminished the likelihood of a diagnosis of retroperitoneal fibrosis [2,3,6].

Sclerosing mesenteritis is an uncommon, idiopathic fibroinflammatory condition affecting the mesenteric adipose tissue, characterized by variable histological components including fat necrosis, chronic inflammation, and fibrosis. Its clinical course is heterogeneous, ranging from asymptomatic radiological findings to chronic abdominal pain, gastrointestinal disturbances, and, less frequently, vascular or obstructive complications. The diagnostic process is often prolonged due to nonspecific symptomatology and radiological findings that resemble those of malignant, infectious, or autoimmune conditions.

In the present case, diagnosis required multiple imaging studies and ultimately a laparoscopic biopsy, as less invasive sampling yielded inconclusive results. This reflects findings in the literature where histological confirmation is often delayed, and non-diagnostic percutaneous or endoscopic biopsies are common in the context of extensive fibrosis [1-3]. According to the American Gastroenterological Association (AGA) 2025 guideline, sclerosing mesenteritis should be evaluated using a stepwise diagnostic approach: initial imaging with CT to identify characteristic features such as mesenteric fat stranding, pseudocapsule, retraction, and calcifications, followed by histopathological confirmation. In this patient, CT and PET-CT imaging were highly suggestive, yet histological confirmation was necessary, illustrating the guideline's recommendations in clinical practice [3].

Of particular note in this case was the progressive involvement of the SMA, culminating in clinical suspicion of vascular compromise. Although rare, fibrotic retraction leading to arterial or venous compression has been documented in the literature, and the AGA guideline specifically recommends vigilant monitoring for vascular or obstructive complications and advocates a multidisciplinary approach when such involvement occurs [3]. The chronicity of the disease, the worsening postprandial pain, and imaging evidence of SMA narrowing warranted referral to vascular surgery, where endovascular and surgical options were evaluated.

Therapeutic management in sclerosing mesenteritis remains largely empirical and individualized. Corticosteroids are commonly used and have demonstrated partial clinical efficacy in several case series [2,3,5]. The AGA guideline recommends a stepwise management strategy, ranging from observation in asymptomatic patients to anti-inflammatory therapy, including corticosteroids, for symptomatic disease, with consideration of additional immunosuppressants or tamoxifen in refractory or progressive cases [3]. In this patient, cyclical oral corticosteroids provided partial relief, with adjunctive therapy including rifaximin for diarrhea and dietary modifications to support gastrointestinal function [3].

An extensive differential diagnosis was considered and systematically excluded through laboratory, imaging, microbiological, and histopathological investigations. These included lymphoma, carcinoid tumor, tuberculosis, sarcoidosis, IgG4-related disease, and retroperitoneal fibrosis. The integration of clinical, radiological, and pathological data was critical for establishing the diagnosis and excluding alternative etiologies.

Numerous case reports and series over the past decade have underscored the complexity of sclerosing mesenteritis, highlighting the value of a multidisciplinary approach, long-term follow-up, and awareness of rare but serious complications, such as vascular compression. Furthermore, the prolonged, often iterative diagnostic process in sclerosing mesenteritis, involving multiple imaging studies, invasive biopsies, and multidisciplinary evaluations, may have significant economic implications, underscoring the potential value of early recognition and guideline-based, stepwise assessment to improve cost-effectiveness in patient management. A summary of the key published reports is presented in Table 2.

**Table 2:** Summary of key publications on sclerosing mesenteritis AGA: American Gastroenterological Association, CT: computed tomography, SM: sclerosing mesenteritis

Authors/year	Study type	Main findings	Clinical relevance
Sharma et al. (2017) [1]	Systematic review (192 cases)	Male predominance; abdominal pain; 8.5% malignancy association	Epidemiological context
Akram et al. (2007) [2]	Retrospective cohort (92 patients)	35% responded to steroids; 42% required surgery	Treatment outcomes
Danford et al. (2019) [4]	Narrative review	Imaging often mimics malignancy; histology essential	Diagnostic challenges
Worthington et al. (2025) [3]	Clinical practice update: AGA guideline	CT-based diagnostic criteria defined by five radiological signs; stepwise management including surveillance, anti-inflammatory agents, and corticosteroids	Evidence-based diagnostic and therapeutic algorithm
Saha et al. (2024) [5]	Concise clinical review	Summarised pathophysiology, imaging features, histological findings, and treatment options in a practical format	Clinically oriented diagnostic and management overview
Rodrigues et al. (2024) [6]	Case report	SM presenting as acute intestinal obstruction	Acute complication management
Gögebakan et al. (2013) [7]	Matched-pair cohort study	Assessed paraneoplastic potential; reported structural compression findings in selected cases	Improved understanding of pathophysiological and diagnostic context
Eze and Halligan (2022) [8]	Review article	Described variable clinical presentations; outlined key CT features, including fat ring sign and pseudocapsule; proposed diagnostic approach	Clarified differential diagnosis and imaging-based decision-making

## Conclusions

Sclerosing mesenteritis is a rare and often under-recognized fibroinflammatory condition that poses significant diagnostic challenges due to its nonspecific symptoms and radiological overlap with malignancy. This case underscores the importance of histopathological confirmation, the limitations of non-invasive biopsy, and the need for multidisciplinary evaluation. The unusual progression to SMA compression highlights the value of long-term surveillance. Management should be individualized, and clinical awareness remains essential for timely diagnosis and appropriate intervention.
